# The mediating role of impulsivity between sleep quality and suicidal ideation in adolescent population: a multicenter cross-sectional study in the northeastern Sichuan, China

**DOI:** 10.3389/fpsyt.2024.1301221

**Published:** 2024-01-29

**Authors:** Yunling Zhong, Jinlong He, Jing Luo, Jiayu Zhao, Yu Cen, Yuhang Wu, Yuqin Song, Cen Lin, Lu Pan, Jiaming Luo

**Affiliations:** ^1^ Mental Health Center, Affiliated Hospital of North Sichuan Medical College, Nanchong, Sichuan, China; ^2^ Department of Neurology, Affiliated Hospital of North Sichuan Medical College, Nanchong, Sichuan, China; ^3^ School of Psychiatry, North Sichuan Medical College, Nanchong, Sichuan, China; ^4^ Department of Psychiatry, Nanchong Psychosomatic Hospital, Nanchong, Sichuan, China

**Keywords:** adolescent, suicidal ideation, sleep quality, impulsivity, adolescence

## Abstract

**Introduction:**

Suicidal ideation is a critical early stage in the progression towards suicidal be havior. Prior research has established links between sleep quality, impulsivity, and suicidal tendencies, yet the interaction among these factors has been less explored. This study aims to explore the mediating role of impulsivity in the relationship between sleep quality and suicidal ideation in adolescents.

**Methods:**

Employing a cross-sectional study design, 6,974 questionnaires were distributed,including the Socio-demographic Characteristics Questionnaire, Barratt Impulsiveness Scale, the Positive and Negative Suicide Ideation Inventory,and the Pittsburgh Sleep Quality Index Scale. The participants were high school and middle school students from 33 schools in northeastern Sichuan, China, selected through random cluster sampling.

**Results:**

Of these 6,786 questionnaires were analyzed. The participant distribution included 47.2% male and 52.8% female students, with 68.3% from junior schools and 31.7% from senior schools. The prevalence of suicidal ideation was found to be 13.6%. The analysis, which involved correlation analysis and the construction of a structural equation model, revealed that sleep quality had a significant positive effect on impulsivity (β:0.289,p < 0.05), and impulsivity, in turn, had a positive impact on suicidal ideation (β:0.355,p < 0.05).Moreover, sleep quality was directly linked to suicidal ideation (β:0.208,p < 0.05). Thus, sleep quality affects suicidal ideation both directly and indirectly through impulsivity.

**Discussion:**

The results of this study suggest that both sleep quality and impulsivity are significant direct influencers of suicidal ideation among adolescents in the region studied, with impulsivity also playing an indirect role in the relationship between sleep quality and suicidal ideation.

## Introduction

Suicidal behavior is a global public health issue, involving physical, psychosocial, and social aspects, including suicidal ideation, suicide attempts, and incomplete suicide. Approximately 703,000 individuals die by suicide each year, and for every completed suicide, there are 20 instances of ideation and attempts at the same time (1). Suicidal behavior is the fourth leading cause of death among adolescents aged 15-19. Alarmingly,1 in 7 adolescents with psychiatric issues do not receive timely assistance (2). Given the substantial burden, effective suicide prevention is crucial. Current theoretical models, like the Three-Step Theory of Suicide(3ST),it is developed by Klonsky’s team, uses the ideation-to-action framework, provides a concise and testable model of suicide, identify the development of suicidal ideation as the first step in suicidal behavior, the higher the intensity of suicidal ideation, the greater the likelihood of suicide (3). The Interpersonal Theory of Suicide suggests that individuals first experience ideation, acquire the capability to commit suicide, and ultimately engage in the act (4). Thus, mitigating ideation is vital for youth suicide prevention. Suicidal ideation refers to the formation of suicide-related thoughts (5), occurring when an individual seriously contemplates ending their life but has not yet attempted or planned to do so (6). In a large-scale global analysis,6-9% of people with suicidal ideation attempt suicide (7). A meta-analysis found that the detection rate of ideation among Chinese adolescents from 2009-2018 was 16.3%,with an 18% suicide attempt rate among those with ideation (8). A cross-sectional study reported a suicidal ideation detection rate of 9.11% among children and adolescents. The rates varied by educational level, with 6.83% in primary school, 10.85% in junior high school, and 16.64% in senior high school (9). These percentages have not shown a significant decline compared to data from the previous decade (10). Implementing early intervention strategies aimed at suicidal ideation is vital for enhancing the effectiveness of suicide prevention efforts.

Sleep quality is a significant issue among the youth population, and it tends to decline as the school year progresses in China (11). In the US, 2/3 of adolescents do not get enough sleep, and sleep quality problems are associated with numerous physical health issues (12). Sleep quality can increase the risk of suicide (13). Some studies have linked sleep deprivation, sleep medication use, and suicidal ideation after controlling for symptoms of depression and anxiety (14). Additionally, both persistent and episodic insomnia problems are associated with increased suicidal ideation (15). Researchers have also suggested a dose-response relationship between sleep quality and suicidal ideation and attempts (16). Sleep may even be a target for suicide prevention; however, the mechanisms of how sleep quality affects suicidal ideation remain unclear and warrant further investigation.

Additionally, beyond sleep quality, impulsivity emerges as another significant factor in suicide risk (17). Impulsivity is characterized by actions undertaken without delay, reflection, voluntary direction, or apparent control in response to stimuli. It plays a crucial role in various adverse behaviors, including those related to suicidal tendencies. Prior research has explored the association between sleep quality and impulsivity, uncovering a connection that involves prefrontal function and its relation to both sleep and impulsivity. They suggest that sleep deprivation and sleep rhythm disturbances impair daytime and nighttime prefrontal function; while, the reduced prefrontal activity may diminish problem-solving ability, and therefore, increase impulsivity and suicide risk (16). One study comparing the effects of impulsivity on self-violence and violence by others showed that, in men, the effect on self-harm is greater (18). Klonsky proposes that impulsivity is a predictor of suicidal ideation (3). A study in China demonstrates that impulsivity is a predictor of suicidal ideation in adolescents (19), with a particular significant effect on explicit suicidal ideation (20). Impulsivity related to emotion is also a predictor, and it mainly affects the degree of ideation (21). However, some studies have also found that impulsive personality traits are distal risk factors for suicidality and are associated with fluctuations in suicidal ideation, independent of suicidal ideation itself (22). Daily variability in both the suicidal ideation and impulsivity status are prospectively related (23). This suggests that the mechanism of impulsivity’s influence on suicidal ideation remains controversial and requires further clarification.

In conclusion, there has been limited exploration of the relationship between sleep quality and impulsive behavior in adolescent populations, particularly regarding their combined impact on suicidal ideation. The specific mechanism through which sleep quality influences suicidal ideation remains unclear. Current research posits the hypothesis that higher levels of impulsivity and poorer sleep quality are likely correlated with suicidal ideation in adolescents. Furthermore, it is conjectured that impulsivity may mediate the relationship between sleep quality and suicidal ideation. This study aims to examine the roles of impulsivity in the nexus between sleep quality and suicidal ideation, utilizing structural equation modeling. The findings of this research are anticipated to provide a theoretical foundation for the early prevention of suicidal behavior in adolescents.

## Methods

### Participants and quality control

Utilizing a cross-sectional research method, a random cluster sampling was conducted to select adolescents from high school and middle school classes in 33 schools in Nanchong, Sichuan Province, China, between November 2021 and May 2022.To ensure data quality, all researchers underwent training in professional knowledge and questionnaire completion. The questionnaires were distributed in classes, with researchers and classroom teachers collaboratively guiding and monitoring the completion process. Researchers conveyed the survey’s purpose to adolescents and their parents at the site, stressing the confidentiality of responses and the adherence to principles of voluntariness and anonymity. Informed consent was obtained through an intelligent platform, with forms being signed by both participants and their parents. The questionnaire included an informed consent form at its outset, and access to the survey was conditional upon both parents and adolescents signing the form on the platform and clicking the agreement button. Adolescents completed the online questionnaire, which underscored that the data would be used solely for scientific research purposes and should be answered honestly. Out of the 6,974 distributed questionnaires 6,786 were included in the final analysis after eliminating responses with missing values, apparent contradictions, and fictitious data. All study procedures adhered to international ethical standards, including the Declaration of Helsinki as revised in 2008 and received approval from the Ethics Committee of Chuanbei Medical College (project number NSMC [2021] 53). The study was also reviewed by the Chinese Clinical Trials Registry (registration number ChiCTR2200058160).

### Measures

Socio-demographic Data:A comprehensive survey was administered using a custom-designed questionnaire by the present investigator, encompassing various parameters such as gender (male or female),educational level (middle school or high school),experience of being left-behind [referring to a demographic group of minors who remain in rural regions, predominantly under the care of extended family members, while their parents migrate to urban areas in pursuit of employment opportunities (24)], sibling status (only child, older brother, older sister, younger brother, or younger sister),living arrangements (living with parents, living with others, living with mother, living with father, living with grandparents),residential environment (urban or rural),school lodging situation (residing on campus or commuting),parental marital status (undivorced, divorced (with father’s custody),divorced(with mother’s custody),or other),long-term medication usage, and other pertinent socio-demographic factors.

The Barratt Impulsiveness Scale (BIS-11):The revised Chinese version of the BIS-11,adapted by Li Shuiyuan Xiao’s team (25), was employed for data collection. This instrument comprises 30 items,subdivided into three dimensions:non-planning, cognitive,and motor impulsiveness,each containing 10 items (e.g.,”I arranged everything carefully”).Each item offers five response options, ranging from ‘never’ to ‘always,’ with corresponding scores from 1 to 5.Reverse scoring is applied to the non-planning and cognitive dimensions.The total score, ranging from 0 to 100,is calculated using the provided formula, with higher scores denoting greater impulsivity. In this study, the McDonald’s omega (ω) reliability coefficient for the BIS-11 was determined to be 0.951.

The Positive and Negative Suicide Ideation Inventory (PANSI):The questionnaire was developed by Osman’s team (26, 27), and Chinese version of it was used for this study. The scale consists of 14 questions, divided into positive (6 questions) (e.g.,”felt confident about your plans for your future?”) and negative dimensions (8 questions) (e.g.,”flet so lonely or sad you wanted to kill yourself so that you could end your pain?”). Each question has 5 options: never, rarely, sometimes, often, and always, with scores ranging from 1 to 5.The higher the total score, the greater the degree of suicidal ideation. In this study, the McDonald’s omega (ω) coefficient for the scale under discussion was 0.918.

The Pittsburgh Sleep Quality Index (PSQI),developed by Buysse and his team in 1993 (28), comprises 19 self-rated and 5 other-rated questions (e.g., “Have you had insufficient energy to do things in the past month?”). The 19th question, along with all five other-rated questions, are not included in the scoring. This scale encompasses seven dimensions: sleep onset, sleep duration, sleep quality, sleep efficiency, sleep disorders, use of hypnotic drugs, and daytime dysfunction. The total score ranges from 0 to 21, with higher scores indicating poorer sleep quality. In this study, the McDonald’s omega (ω) coefficient for the PSQI was determined to be 0.764.

### Sample size calculation

This cross-sectional study required a two-sided test with a test level *α* of 0.05 and a tolerance error *δ* of 0.01.The predicted incidence of suicidal ideation among adolescents is 20% according to the literature (29). Using PASS15 software, the minimum sample size of 6245 cases was calculated. Considering that 10% of the respondents would lose data or refuse to complete the study, we adjusted the minimum sample size to 6938 cases.

### Statistical analysis

We utilized SPSS Statistics 26.0 to conduct a descriptive statistical analysis, aiming to elucidate the distribution characteristics of the sample. In this cohort, males constituted 47.2%, while females made up 52.8%. Among these participants, 68.3% were junior high school students, and 31.7% were senior high school students. The detected rate of suicidal ideation in this population was 13.6%. To ascertain whether there were any notable differences in impulsivity and sleep quality between adolescents with and without suicidal ideation, we conducted a comparative analysis. This involved using the Mann-Whitney U test for continuous variables to compare the two groups. The results of this analysis are presented in **Tables 1** and **2**. Furthermore, to investigate the relationships between the primary variables, a Spearman correlation analysis was conducted. For structural equation modeling, we utilized Amos 26.0, which comprises of two components - the measurement model and the structural model. The measurement model portrays the relationship between latent and observed variables, while the structural model represents that between latent variables. In this study, the latent variables included suicidal ideation, impulsivity, and sleep quality. Confirmatory factor analysis was employed to assess if the factor structure could be fitted to the data. To test our hypotheses and evaluate the structural equation model fit, we utilized the Maximum Likelihood (ML) method to examine the path coefficients and the overall model.

**Table 1:** Demographic characteristics of the participant.

		Frequency	Percentage
Gender	Female	3203	47.20%
	Male	3583	52.80%
Educational level	Junior High School	4636	68.30%
	Senior High School	2150	31.70%
Experience of being left-behind	No	2799	41.20%
	Yes	3987	58.80%
Living arrangements	living with parents	2036	30.00%
	living with others	435	6.40%
	living with mother	1466	21.60%
	living with father	317	4.70%
	living with grandparents	2532	37.30%
Parental marital status	Undivorced	4983	73.40%
	Divorced(with father’s custody)	825	12.20%
	Divorced(with mother’s custody)	220	3.20%
	Other	758	11.20%
Residential living environment	Urban areas	2082	30.70%
	Rural areas	4704	69.30%
School lodging situation	Commuting	2733	40.30%
	Residing on campus	4053	59.70%
Sibling status	Only child	1007	14.80%
	With older brother	1785	26.30%
	With older sister	1815	26.70%
	With younger brother	1155	17.00%
	With younger sister	1024	15.10%
Long-term medication usage	No	6592	97.10%
	Yes	194	2.90%
Suicidal ideation	No	5866	86.40%
	Yes	920	13.60%

**Table 2:** Comparison of sleep quality and impulsivity.

	Suicidal ideation group	Non-suicidal ideation group	*Z*	*P*
Sleep quality	6.0(4.0,9.0)	4.0(2.0,6.0)	17.5	<0.001
Impulsivity	49.2 (41.7,53.3)	41.7 (29.2,51.7)	12.3	<0.001

## Results

### Demographic characteristics of the participants

We investigated a total of 6,974 students and ultimately collected 6,786 valid questionnaires, with a response rate of 97.3%.The respondents ranged in age from 10 to 19,with 52.8% being male and 68.3% being junior high school students. In this paper,left-behind experience refers to the experience of children aged 16 and below who are left at their household registration location for half a year or more and cannot live with both parents because both parents or one of them go out to work (30). Such experience was reported by 58.2% of the sample. Besides,the incidence of suicidal ideation was 13.6%. According to **Table 2**, the median score of sleep quality for the suicidal ideation group (*P*25,*P*75) was 6.0 (4.0,9.0),while that for the non-suicidal ideation group (*P*25,*P*75) was 4.0 (2.0,6.0). The nonparametric Mann-Whitney U rank sum test was also performed between the two groups, with Z=17.5 and P<0.001, indicating a statistically significant difference in sleep quality scores between them. In addition, the median impulsivity score in the suicidal ideation group (*P*25,*P*75) was 49.2 (41.7,53.3),while that in the non-suicidal ideation group (*P*25,P75) was 41.7 (29.2,51.7). The nonparametric Mann-Whitney U rank sum test between the two groups yielded *Z*=12.3 and *P*<0.001, indicating a statistically significant difference in impulsivity scores between them.

### Structural equation model and correlation analysis

**Figure 1:**
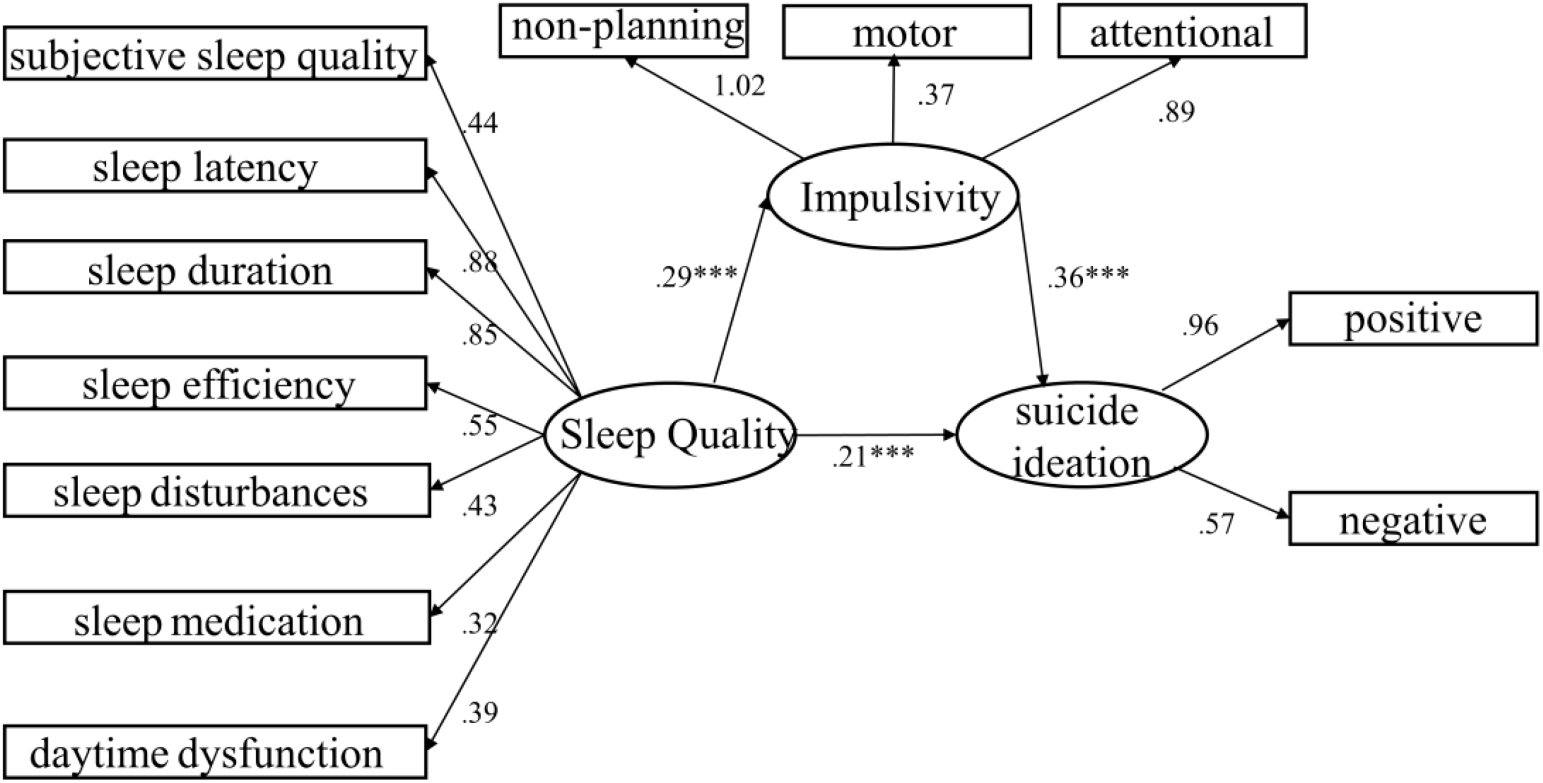
Structural equation model: rectangles:observed variables;circles:unobserved variables. Numbers by single-headed arrows reflect standardized path efficiency. The model fitness indicators:CMIN/DF: 3.64, RMSEA:0.02,SRMR:<0.01,GFI:0.995,CFI:0.996,TLI: 0.995.***p<0.001.

For this study, we utilized the ML method to establish the structural equation model. Indeed, a dataset that complies with multivariate normality is crucial for enhancing the robustness and accuracy of a structural equation model (SEM). Through the computation of kurtosis and skewness, it was determined that the data in this study did not conform to the assumption of multivariate normal distribution. Additionally, in larger sample sizes, cardinality expansion can occur even if multivariate normality is initially met. To rectify this issue, we applied the Bollen-Stine correction method to adjust the fitness indexes (31). Our findings revealed that the cardinality inflation was attributable to the large sample size. After applying the Bollen-Stine correction, all model fitness indicators met the necessary criteria. As illustrated in **Figure 1**, these criteria included:CMIN/DF (excluding the chi-square goodness-of-fit test) at 3.64, RMSEA (root mean square error of approximation) at 0.02, SRMR (Standardized Root Mean Square Residual) less than 0.01,GFI (Goodness of Fit Index) at 0.995, CFI (Comparative Fit Index) at 0.996, and TLI (Tucker–Lewis Index) at 0.995.Moreover,the Cronbach coefficients of the scales utilized in this study were all above 0.9,indicating that the reliability of each scale was good.

**Table 3** demonstrates the correlation between sleep quality, impulsivity, and suicidal ideation. The path relationships of each variable and standardized regression coefficient(β) are shown in **Table 4** and **Figure 1**. The path relationship hypothesis test reveals that sleep quality positively affects impulsivity(β:0.289,*p*<0.001),impulsivity positively affects suicidal ideation (β:0.355,*p*<0.001),and sleep quality positively affects suicidal ideation (β:0.208,*p*<0.001). The construct reliability (C.R) was significant and acceptable(12.932-25.342). The estimates of direct, indirect, and total effects are presented in **Table 5**, sleep quality exerts only direct effects on impulsivity, impulsivity exerts only direct effects on suicidal ideation, while sleep quality exerts both direct and indirect effects on suicidal ideation.

**Table 3:** Spearman correlation analysis.

	suicidal ideation		sleep quality	impulsivity
suicidal ideation	1			
sleep quality	.492**		1	
impulsivity	.646**		.428**	1

**P<0.01.

**Table 4:** Standardized regression coefficient(β).

			Estimate	S.E.	C.R.	P
Impulsivity	<-	Sleep quality	0.289	1.091	19.3	***
Suicidal ideation	<-	Impulsivity	0.355	0.004	25.342	***
Suicidal ideation	<-	Sleep	0.208	0.33	12.932	***

***P<0.001.

**Table 5:** Effects of the structural model.

Paths		Total effect	Direct effect	Indirect effect
Impulsivity	Sleep quality	0.289	0.289	
Suicidal ideation	Sleep quality	0.311	0.208	0.103
	Impulsivity	0.355	0.355	

## Discussion

This study revealed that adolescents with suicidal ideation exhibited poorer sleep quality and heightened impulsivity compared to those without such ideation. Consequently, a structural equation model of the three variables was developed to assess the mediating influence of impulsivity on the relationship between sleep quality and suicidal ideation in adolescents. This research is notably the first to probe the interaction between sleep quality and impulsivity in influencing suicidal ideation among adolescents in northeastern Sichuan,China.It is characterized by its multicenter nature, large sample size, and high questionnaire response rate.

A preliminary comparison found differences in sleep quality and impulsivity between adolescents with suicidal ideation and those without suicidal ideation. To be specific, adolescents with suicidal ideation exhibited poorer sleep quality and higher impulsivity, which was in line with the reality. Despite the bivariate correlation between sleep quality and impulsive behavior revealed by existing researches (32), no evidence demonstrates the influence of such a correlation on adolescent suicidal ideation.Therefore,the reasons for these differences will be further discussed combined with other findings of this study.

The structural equation model in this study substantiates the direct positive influence of sleep quality on adolescent suicidal ideation, aligning with existing research that links poor sleep quality to heightened suicidal ideation the following day (33). Poor sleep quality in adolescents may directly affect suicidal ideation, potentially due to physical fatigue and discomfort resulting from inadequate sleep. This direct effect might also be attributed to uncontrollable factors that contribute to poor sleep quality, such as academic pressures, electronic device use, and other stressors. Thus, further research is needed within the adolescent demographic to understand the mechanisms of sleep’s direct impact on suicidal ideation. However, some studies have found no correlation between sleep quality and suicidal ideation, suggesting that improving sleep quality may not affect suicidal ideation and planning (34). Indeed, research in this field has yielded varied findings regarding the potential link between sleep quality and suicidal ideation. This discrepancy may arise from the mediating role of impulsivity in the relationship between sleep quality and suicidal ideation, a factor not considered in some studies where impulsivity was not included as a variable. Integrating the results of this study, it is concluded that impulsivity is a critical factor in the relationship between sleep quality and suicidal ideation. Additionally, sleep quality may heighten the level of suicidal ideation by influencing impulsivity. Thus, the connection between sleep quality and suicidal ideation may weaken in the absence of impulsivity’s influence.

This study proves the indirect positive effect of sleep quality on suicidal ideation through impulsivity,which supports the above hypothesis.Previous studies mentioned that sleep disruption led to increased impulsivity among adolescents with bipolar disorder (35). Moreover,the association between sleep quality and suicidal ideation is significant as to patients with psychiatric disorders (36). In particular,when it comes to individuals with borderline personality disorder,poor sleep quality is significantly correlated with self-reported impulsivity,which is immune from other co-morbidities (37). These findings are consistent with this study.More importantly,adolescents are in a period of unstable physiological and psychological development,where impulsivity may present a dynamic process as the nervous system matures (38). Additionally,sleep is vital for neurodevelopment (39). Current studies that focus on the impulsivity genome in adolescents find that genetic susceptibility to impulsivity is a predictor of suicidal ideation (40) while poor sleep quality affects the neural function of brain regions governing social-emotional processing, leading to decreased impulse control (36). Therefore, sleep quality should be of particular concern for adolescents with genetic susceptibility to impulsivity or borderline personality disorder, whose impulsivity may be more susceptible to sleep,thus highlighting the association between sleep quality and suicidal ideation.Accordingly,timely intervention in the sleep quality of adolescents with high impulsive suicidal ideation can achieve favorable results.The neurophysiological mechanisms of sleep quality and impulsivity on suicidal ideation among adolescents should be further investigated in the future,so as to evaluate the intensity of suicidal ideation from a physiological or imaging perspective and facilitate timely intervention.Besides,the screening of impulsive personality benefits early prevention of suicide (41).

According to this study,impulsivity directly and positively influences the degree of suicidal ideation, and if impulsivity in adolescents continues to increase because of poorer sleep quality, suicidal ideation will gradually increase and evolve into suicidal action.Other studies also point out that impulsive personality traits affect suicidal intention by influencing individual suicidal ideation,and that individuals with high impulsivity and suicidal ideation should be of particular concern (42). In addition, impulsivity may even facilitate the transition from suicidal ideation to action (43), which may be related to pain and pain-stimulating events.Interpersonal suicide theory suggests that impulsivity is linked to the acquisition of suicidal capability through pain and pain-stimulating events (e.g.,non-suicidal self-injurious behavior) (4). Other scholars have linked sleep-induced increases in impulsivity to unplanned suicidal behavior.The difference in views may be related to the fact that suicidal ideation has not yet been evaluated for those who have succeeded in suicide.Therefore,future studies should focus on individualized suicidal ideation screening tools for adolescents with poor sleep quality and high impulsivity,so as to identify and assess suicidal ideation early to avoid suicidal behavior.

This study has several limitations. Firstly, its cross-sectional design precludes the drawing of causal conclusions.The data was collected via questionnaires, which could be subject to recall bias. Additionally, the sample comprises more middle school students than high school students, potentially leading to selection bias. Another limitation is the lack of data on non-binary gender identities, which may affect the generalizability and applicability of the findings. Furthermore, the study does not encompass other potentially influential factors, such as non-suicidal self-injury behavior, depression, and various personality traits, all of which might directly impact sleep quality, impulsivity, and suicidal ideation (44, 45). To avoid overfitting and maintain model simplicity, this study deliberately excluded depression as a variable in the structural equation model. While previous research has established the influence of depression on sleep quality and suicidal ideation, its relationship with impulsivity requires further investigation. This exclusion of depression and other variables is a significant limitation of this study. Future research should therefore conduct more comprehensive analyses, including a broader range of variables and a more segmented examination of the adolescent population, to more fully explore these relationships.

In conclusion, this study has validated the crucial role of impulsivity in the relationship between sleep quality and suicidal ideation among Chinese adolescents aged 10-19.It highlights the significant direct effect of impulsivity on suicidal ideation. These findings reaffirm the importance of sleep quality in the psychological well-being and personality development of adolescents. Furthermore, the study draws attention to the need for clinicians,parents,and educators to be vigilant about impulsive traits in adolescents. Adolescents demonstrating impulsive behaviors should be screened for symptoms associated with poor sleep quality and provided with early interventions. These insights pave new avenues for suicide prevention strategies among adolescents. Looking forward, future research should delve into the neurophysiological mechanisms underlying how sleep quality influences suicidal ideation through impulsivity,which will enhance the development of individualized approaches to suicide prevention.

## Data availability statement

The raw data supporting the conclusions of this article will be made available by the authors, without undue reservation.

## Ethics statement

All study procedures conformed to international ethical standards, the Declaration of Helsinki revised in 2008, and were approved by the Ethics Committee of Chuanbei Medical College (project number NSMC [2021] 53). All the students and their guardians who participated in this study fully understood all the contents of this study, and expressed their willingness to participate in this study, and signed informed consent.

## Author contributions

YZ: Conceptualization, Data curation, Formal analysis, Methodology, Validation, Writing – original draft, Writing – review & editing. JH: Conceptualization, Data curation, Formal analysis, Methodology, Validation, Writing – original draft, Writing – review & editing. JL: Data curation, Investigation, Methodology, Validation, Writing – original draft. JZ: Data curation, Investigation, Methodology, Validation, Writing – original draft. YC: Data curation, Investigation, Supervision, Validation, Writing – review & editing. YS: Data curation, Investigation, Supervision, Validation, Writing – review & editing. YW: Data curation, Investigation, Supervision, Validation, Writing – review & editing. CL: Data curation, Investigation, Supervision, Validation, Writing – review & editing. LP: Data curation, Investigation, Supervision, Validation, Writing – review & editing. JML: Conceptualization, Funding acquisition, Methodology, Project administration, Resources, Software, Supervision, Writing – review & editing.
